# Assessment of the Trabecular Bone Microstructure Surrounding Impacted Maxillary Canines Using Fractal Analysis on Cone-Beam Computed Tomography Images †

**DOI:** 10.3390/diagnostics14192143

**Published:** 2024-09-26

**Authors:** Ezgi Sunal Akturk, Ahsen Irem Toktas, Erkay Can, Ezgi Kosen, Irfan Sarica

**Affiliations:** 1 Department of Orthodontics, Hamidiye Faculty of Dental Medicine, University of Health Sciences, Istanbul 34668, Turkey; 2 Department of Orthodontics, Faculty of Dentistry, Bezmialem Vakif University, Istanbul 34093, Turkey; 3 Faculty of Dentistry, Bezmialem Vakif University, Istanbul 34093, Turkey; 4 Department of Orthodontics, Institute of Health Sciences, Bezmialem Vakif University, Istanbul 34093, Turkey; 5 Department of Oral and Maxillofacial Radiology, Faculty of Dentistry, Bezmialem Vakif University, Istanbul 34093, Turkey

**Keywords:** bone density, cone-beam computed tomography, fractal dimension, impacted maxillary canines

## Abstract

Objectives: To assess the impact of the presence or position (buccal/palatal) of impacted canines on trabecular bone density using fractal analysis (FA) on cone-beam computed tomography (CBCT) images, and to compare the results with a control group without impacted canines. Methods: This retrospective study included 41 patients with unilateral impacted canines (30 palatal, 11 buccal) and a control group of 39 patients who underwent surgically assisted rapid maxillary expansion. All patients had CBCT images recorded for diagnostic and treatment purposes. Cross-sectional CBCT images were obtained between the first and second premolars on both sides of the patients’ maxilla. From these images, fractal dimension (FD) was measured in a 20 × 20 pixel region of interest in the trabecular bone using the ImageJ software. Results: The FD values were significantly higher on the impacted side in the impacted canine group (*p* = 0.02). Within the impacted canine group, a significant increase in FD was observed on the impacted side in the buccal-impacted subgroup (*p* = 0.02), while no significant difference was observed in the palatal-impacted subgroup (*p* > 0.05). Conclusions: According to the results of our study, there is an association between the position of the impacted canine and trabecular bone density. An increased trabecular bone density may play a role in the etiology of buccally impacted canines. Clinicians should consider anchorage planning, and appropriate force level, during the forced eruption of buccally impacted canines with high surrounding bone density, to minimize undesirable movements and achieve optimal treatment outcomes.

## 1. Introduction

Impacted teeth are one of the most common problems encountered in orthodontic clinics. After third molars, maxillary canines have the second highest prevalence of impaction, with a rate of 1–5% [1,2,3]. Approximately 8% of all impacted maxillary canines are bilateral, with about one-third of the remaining unilateral impactions occurring in the buccal position, and the remaining two-thirds in the palatal position [4,5].

Canine impaction is known to be associated with various factors such as genetics, early loss or persistence of primary canines, and abnormally positioned permanent canines [6]. Maxillary lateral incisor agenesis is a common cause of palatal impaction, while crowding is more often associated with buccal impaction [7,8]. Recently, researchers have also been investigating the effect of bone density as a local factor in canine impaction [9,10,11].

Radiographic methods are effectively used for the diagnosis of impacted teeth. Radiographic diagnosis can determine the position of impacted teeth, their relationship with adjacent structures, and treatment options [12]. While conventional radiographs such as panoramic, periapical, and occlusal films are useful for diagnosis, they are not as detailed as three-dimensional techniques. Computed tomography (CT) can be used for more detailed examinations, but the high radiation dose and cost limit its use. Cone-beam computed tomography (CBCT) is used in dental practice to provide three-dimensional data with lower radiation doses and costs compared to CT [13]. CBCT is reported to give more accurate results in the diagnosis of impacted maxillary canines compared to conventional radiographs [14]. However, few studies have evaluated maxillary bone characteristics around impacted and nonimpacted contralateral canines using CBCT [9,10].

Fractal analysis is a mathematical tool used as one of the various methods of assessing trabecular bone structure in the fields of medicine and dentistry. The box-counting method is the most widely used technique for estimating the fractal dimension (FD) [15]. Fractal analysis allows the measurement of irregular and complex structures like the maxillary bone [16,17]. FD values obtained from CBCT scans have been associated with bone mineral density [17] and trabecular bone pattern [18]. Low FD values indicate larger gaps in the trabecular structure, while high FD values indicate greater density and complexity of the trabecular bone [19].

It has been reported that bone density is affects orthodontic tooth movement, with the rate of movement increasing as the bone density reduces. This is evidenced by the observation that tooth movement occurs more rapidly in children than in adults [20,21]. Based on this information, the idea that bone density could affect the prognosis of treatment during the orthodontic forced eruption of impacted canines prompted us to conduct this study.

The aim of this study was to compare bone density between the impacted and fully erupted sides in patients with unilateral maxillary impacted canines using fractal analysis of CBCT images, and to assess the relationship between bone density and the position of the impacted canines (buccal/palatal). The null hypothesis was that bone density does not differ between the impacted and nonimpacted sides, and is not based on the buccal or palatal position of the impacted canine.

## 2. Materials and Methods

This retrospective study was approved by the Noninvasive Clinical Trials Ethics Committee of Bezmialem Vakif University (approval no: 2022/43, 22 February 2022). The study sample consisted of patients aged 13 to 25 years who presented to the orthodontics department of the Bezmialem Vakif University Faculty of Dentistry between January 2015 and July 2024 and had CBCT data on file. A power analysis indicated that a minimum of 36 individuals per group would be required to achieve 80% power for detecting significant differences with an effect size of 0.60 at a significance level of α = 0.05 [9].

A total of 134 patients with maxillary impacted canines who had CBCT images in the archive were assessed for eligibility.

The inclusion criteria were as follows:

- Availability of CBCT images of unilateral maxillary impacted canines.
- Complete eruption of the contralateral canine.
- Absence of systemic or genetic diseases affecting bone metabolism.
- No orthodontic treatment history.

Exclusion criteria were as follows:

- CBCT scans acquired at a different clinic.
- Congenitally missing or extracted teeth other than third molars.
- Presence of supernumerary teeth or any cystic formations in CBCT.
- Presence of artifacts on CBCT images.
- History of dentoalveolar trauma.

Forty-nine patients meeting these criteria were included in the study. Eight patients without the required CBCT sections were later excluded. The final impacted canine group comprised two subgroups: 30 patients with palatal- (23 females, 7 males) and 11 with buccal- (7 females, 4 males) impacted maxillary canines.

The control group consisted of 39 patients (22 females, 17 males) who had undergone surgically assisted rapid maxillary expansion (SARME) treatment at the same university, had no systemic or genetic diseases affecting bone health, had no missing teeth except for third molars, and had CBCT images on file.

The patients’ CBCT images were used to measure FD values around the maxillary canines and to examine the positions of the impacted canines. All CBCT images were acquired using the Planmeca ProMax 3D Mid (Planmeca, Helsinki, Finland) tomography device, with parameters set to an average of 90 kV, 10 mA, using an 8 × 8 cm FOV, 18 s exposure time and 0.2 mm voxel size. Similar to Servais et al. [9], cross-sectional CBCT images between maxillary first and second premolars were obtained by same dentomaxillofacial radiologist with 5 years of experience (İ.S.).

**Figure 1:**
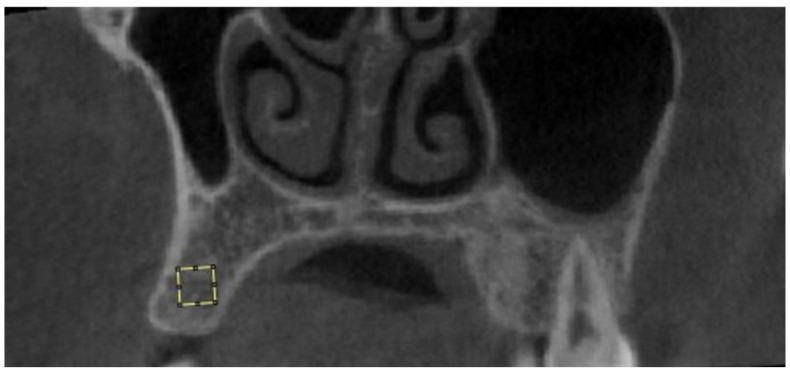
Selection of 20 × 20 pixel ROI on a CBCT image.

**Figure 2:**
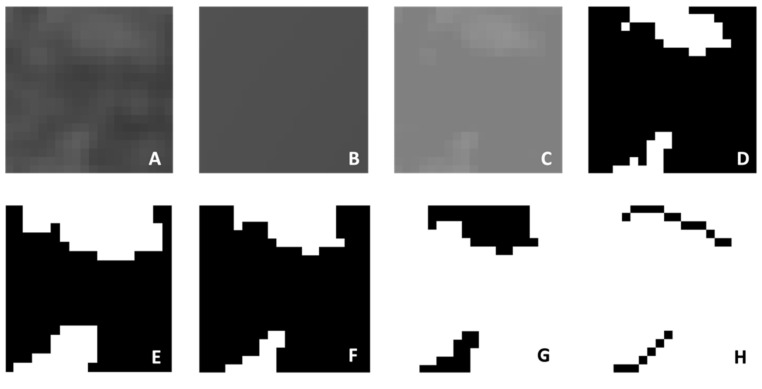
Stages of fractal analysis: (**A**) duplicated image; (**B**) blurred with a Gaussian filter; (**C**) addition of 128 gray values to each pixel; (**D**) binarized into a two-color image; (**E**) eroded; (**F**) dilated; (**G**) inverted; (**H**) skeletonized.

The obtained images were saved in JPEG format using Planmeca Romexis (version 3.8.3.R, Planmeca, Helsinki, Finland). Fractal analysis was performed using the box-counting method developed by White and Rudolph [22] in the ImageJ software version 1.53a (National Institutes of Health, Bethesda, MD, USA). All analyses were conducted by the same researcher (E.C.) on the same computer, using a 27-inch flat panel display with a resolution of 1920 × 1080 pixels (Samsung Electronics, Dublin, Ireland). The researcher was trained by İ.S., who has experience in fractal analysis. A 20 × 20 pixel region of interest (ROI) was selected avoiding roots, cortical bone, and vascular canals (Figure 1) [11]. JPEG images of the ROI were duplicated into two images, and a Gaussian filter (Sigma = 35 pixels) was applied to blur the images, for the purpose of eliminating fine- and medium-scale variations in image brightness. The blurred image was then subtracted from the original image. A value of 128 was added at each pixel location to discriminate bone marrow spaces from trabeculae. The resulting image was binarized into a two-color image, black and white, with a threshold value of 128. To reduce noise, the image was first eroded, then dilated. To make the trabeculae black and the marrow spaces white, the image was inverted. Finally, the image was skeletonized (Figure 2). The FD of the skeletonized image was calculated using the “Fractal Box Counting” option in the ImageJ software version 1.53a (National Institutes of Health, Bethesda, MD, USA).

### Statistical Analysis

FD measurements were repeated by the same investigator (E.C.) for ten randomly selected patients from both groups after 2 weeks, in order to determine intra-observer reliability. To assess inter-observer reliability, a second investigator (İ.S.) measured FD from the same CBCT images. The intraclass correlation coefficient (ICC) was calculated to analyze the intra- and inter-observer reliability. The normality of the data was assessed using the Shapiro–Wilk test. Age and sex distributions were compared between the groups using Mann–Whitney U Test and Chi-square Test, respectively. Intragroup comparisons were performed using the independent-samples *t*-test. Intergroup comparisons involved calculating the differences in FD values between impacted and nonimpacted sides within the impacted canine groups, as well as between the right and left sides in the control group. The absolute values of these differences were compared between groups using the Kruskal–Wallis Test. Statistical analysis was conducted using the SPSS statistics software, version 22.0 (IBM Corp., Armonk, NY, USA). A statistical significance level of *p* < 0.05 was used for each comparison.

## 3. Results

ICC values for the FD measurements ranged from 0.85 to 0.94, indicating excellent—intra-observer reliability, and from 0.88 to 0.92, indicating excellent inter-observer reliability.

There was no significant difference in sex distribution between the groups (*p* = 0.16). The mean age of individuals in the control group was significantly higher than that in the impacted canine group (*p* < 0.001) (Table 1).

Comparison of the FD values between the impacted and nonimpacted sides in the 41 patients with unilateral maxillary impacted canines showed that the FD values on the impacted side were significantly higher than those on the nonimpacted side (*p* = 0.02). Within the impacted canine subgroups, a significant increase in FD was observed on the affected side in the buccal-impacted subgroup (*p* = 0.02), while there was no significant difference between the sides in the palatal-impacted subgroup (*p* > 0.05). There was also no statistically significant difference in the FD values on the right and left sides of individuals in the control group (*p* = 0.22) (Table 2).

To make intergroup comparisons, we determined the absolute difference in FD between the impacted and nonimpacted sides for patients with impacted canines and between the right and left sides for the control group. Comparisons of these values among the buccal-impacted canine group (*n* = 11), palatal-impacted canine group (*n* = 30), and control group (*n* = 39) revealed no statistically significant difference (*p* = 0.78) (Table 3).

## 4. Discussion

Various radiographic methods are used to examine bone quality. Micro-CT is considered the gold standard for evaluating bone morphology and microstructure [23]. However, its high cost, radiation exposure, and need for ex vivo bone samples hinder its use [15]. CT is a method that uses calibrated Hounsfield unit (HU) values to assess bone density [20]. However, CBCT is often preferred in dental clinical practice due to its advantages of low cost, reduced radiation dose, and minimal image distortion [24]. Many studies have reported a high correlation between grayscale values obtained from CBCT and HU, suggesting the potential of CBCT for assessing bone density [25].

Fractal analysis is another method used to quantitatively measure bone quality and microstructure. This method uses statistical texture analysis to examine the trabecular bone micro-architecture, with the numerical expression FD used as a measure of image complexity [26]. In an in vitro study, Southard et al. [19] reported a high correlation between FD and the density of alveolar bone. Additionally, previous studies have shown that FD values calculated from CBCT scans are associated with bone mineral density and trabecular pattern [17,18]. While the fractal analysis method offers advantages such as independence from variables like radiation dose, ease of accessibility, and applicability, its disadvantages include susceptibility to the location of the selected ROI, making it challenging to assess trabecular bone with linear ROI [27]. In our study, FD was calculated from routine CBCT images obtained for the purpose of determining the position of impacted canines, in order to examine the trabecular bone microarchitecture.

When comparing age and sex between the impacted canine and control groups in our study, there was no statistically significant difference in sex distribution, whereas the control group was older on average than the impacted canine group. We attribute this difference to the inclusion of patients who underwent SARME in the control group, which was selected from among these patients due to the retrospective nature of our study and the routine use of CBCT for surgical planning before SARME.

Patients with bilateral impacted canines were excluded from this study. Our rationale for including only patients with unilateral impacted canines was to use the nonimpacted sides of these individuals as their own controls. When the FD values of the impacted and nonimpacted sides were compared in the impacted canine group, significantly higher FD values were found on the impacted side compared to the nonimpacted side. In contrast, no significant difference was observed when comparing the right and left sides in the control group. Similar studies conducted by Servais et al. [9] and Arvind et al. [10] also examined bone density around impacted maxillary canines using fractal analysis employing four parameters: bone area, bone FD, bone marrow area, and bone marrow FD. Neither of these studies included a control group, and both detected no significant difference in FD values between the impacted and nonimpacted sides. However, they did observe significant differences in bone surface area and bone marrow surface area on the impacted side. Although a significant difference in FD values was not observed between the impacted and nonimpacted sides in these studies, the significant variations in bone and bone marrow surface areas led to the conclusion that there was increased bone density in the impacted canine region. In both studies, the ROI areas were defined as being 64 × 64 pixels in size using the Office Picture Manager program (Microsoft, Redmond, WA, USA, available online https://www.officepicturemanager.com, accessed on 10 August 2024) and the images were subsequently transferred to the ImageJ software (National Institutes of Health, Bethesda, MD, USA, available online https://imagej.net/ij/download.html, accessed on 10 August 2024). In our study, we selected standard 20 × 20 pixel ROI areas directly from cross-sectional CBCT images within the ImageJ software version 1.53a (National Institutes of Health, Bethesda, MD, USA), following a similar approach to that described in a study by Kocak and Bulut [28]. As highlighted in a systematic review [15], variations in ROI size and location can affect FD values. Therefore, the lack of significant differences among FD values in the studies by Servais et al. [9] and Arvind et al. [10] might be related to disparities in ROIs. Koseoglu Secgin et al. [11] evaluated bone density using gray values obtained from CBCT and reported that gray values in the impacted canine area were statistically significantly higher than in the nonimpacted area. They suggested that local increases in bone density could be an etiological factor in canine impaction.

To investigate the relationship between bone structure and the position of impacted canines, we subdivided the impacted canine group based on buccal and palatal positioning. In the buccal impacted canine subgroup, a significant difference in FD values was observed between the impacted and nonimpacted sides, whereas no significant difference was found in the palatal-impacted canine subgroup. This contradicts the findings of Arvind et al. [10], who found no differences in FD values between the impacted and nonimpacted sides in both the buccal and palatal-impacted canine subgroups. However, they also observed a significantly larger bone surface area on the impacted side. In light of these results, the researchers emphasized the possibility that bone density could be a local factor in both buccal and palatal impaction cases. Furthermore, meticulous anchorage planning and the application of optimal force levels were identified as crucial for achieving favorable functional and aesthetic outcomes, particularly when increased bone density is observed in the impacted canine region. They suggested the use of temporary anchorage devices to enhance anchorage and prevent undesirable movement of the reactive units, as well as methods of accelerating tooth movement to reduce bone resistance [10].

Since age distribution was not homogeneous between the groups and could potentially influence bone density, we did not directly compare FD values between the impacted canine group and control group. To account for these variations, we calculated the absolute differences in FD between the impacted and nonimpacted sides in the palatal- and buccal-impacted canine subgroups, as well as between the right and left sides in the control group for inter-group comparison. The difference between the impacted and nonimpacted sides in both the buccal and palatal subgroups was found to be similar to the difference between the right and left sides in the controls.

Limitations of this study include its retrospective nature, small sample size, and higher mean age in the control group. Other limitations are the dependence of fractal analysis on image quality, the difficulty of obtaining sections from the area to be examined, and the presence of the primary canine root or dental follicle of the impacted canines, which makes it difficult to obtain a section directly from the canine tooth area. Further studies with larger sample sizes and control groups of similar age should be conducted to address these limitations.

## 5. Conclusions

According to the findings of our study, individuals with unilateral impacted canines have higher trabecular bone density on the impacted side compared to the nonimpacted side, and the position of the impacted canine is associated with trabecular bone density. The findings suggest that increased trabecular bone density may be among the local factors in the etiology of buccally impacted canines. Clinicians should consider anchorage planning and appropriate force level during the forced eruption of buccally impacted canines with high surrounding bone density to minimize undesirable movements and achieve optimal treatment outcomes.

## Figures and Tables

**Table 1:** Age and sex comparison of the impacted canine and control group.

	Impacted Canine Group (*n*:41)		Control Group (*n*:39)		
**Sex**	*n*	%	*n*	%	***p* value**
Female	30	73.2	22	56.4	0.12
Male	11	26.8	17	43.6	
**Age (year)**	Mean	SD	Mean	SD	***p* value**
	15.79	3.29	19.61	2.38	**<** **0.001** ***

SD, standard deviation; *** *p* < 0.001.

**Table 2:** Intragroup comparison of FD values in impacted canine group, subgroups, and control group.

		Mean	SD	*p* Value
Impacted Canine Group (*n*:41)	Impacted Side	1.02	0.12	**0.02** *
	Nonimpacted Side	0.96	0.12	
Buccal Impacted Canine Group (*n*:11)	Impacted Side	1.07	0.08	0.02 *
	Nonimpacted Side	0.95	0.13	
Palatal Impacted Canine Group (*n*:30)	Impacted Side	1.01	0.12	0.17
	Nonimpacted Side	0.96	0.11	
Control Group (*n*:39)	Left Side	0.97	0.11	0.22
	Right Side	0.94	0.11	

SD, standard deviation; * *p* < 0.05.

**Table 3:** Intergroup comparison of FD values differences between right and left sides.

	Mean Difference	SD	*p* Value
Palatal Impacted Canine Group (*n*:30)	0.13	0.11	0.78
Buccal Impacted Canine Group (*n*:11)	0.14	0.11	
Control Group (*n*:20)	0.10	0.06	

SD, standard deviation.

## Data Availability

The data presented in this study are available on request from the corresponding author.
